# Repair of the pronator quadratus after volar plate fixation in distal radius fractures: a systematic review

**DOI:** 10.1007/s11751-017-0288-4

**Published:** 2017-05-17

**Authors:** Marjolein A.M. Mulders, Monique M.J. Walenkamp, Fernande J.M.E. Bos, Niels W.L. Schep, J. Carel Goslings

**Affiliations:** 1 Trauma Unit, Department of Surgery, Academic Medical Center, University of Amsterdam, P.O. Box 22660, 1100 DD Amsterdam, The Netherlands; 2 Department of Surgery, Maasstad Hospital, P.O. Box 9100, 3007 AC Rotterdam, The Netherlands

**Keywords:** Distal radius, Fracture, Volar plate fixation, Pronator quadratus, Repair

## Abstract

To position the volar plate on the distal radius fracture site, the pronator quadratus muscle needs to be detached from its distal and radial side and lifted for optimal exposure to the fracture site. Although the conventional approach involves repair of the pronator quadratus, controversy surrounds the merits of this repair. The purpose of this study was to compare the functional outcomes of patients with distal radius fractures treated with pronator quadratus repair after volar plate fixation versus no pronator quadratus repair. A systematic search was conducted in Medline, EMBASE and the Cochrane Central Register of Controlled Trials, on 23 July 2015. All studies comparing pronator quadratus repair with no pronator quadratus repair in adult patients undergoing volar plate fixation for distal radius fractures were included. The primary outcome was the Disability of the Arm, Shoulder and Hand (DASH) score at 12 months. Secondary outcomes included range of motion, grip strength, post-operative pain and complications. A total of 169 patients were included, of which 95 underwent pronator quadratus repair, while 74 patients underwent no pronator quadratus repair. At 12 months follow-up no statistically significant differences in DASH-scores and range of motion were observed between pronator quadratus repair and no repair. Moreover, post-operative pain and complication rates were similar between both groups. At 12 months of follow-up, we do not see any advantages of pronator quadratus repair after volar plate fixation in the distal radius. However, a definitive conclusion cannot be drawn from this systematic review due to a lack of available evidence.

## Introduction

Distal radius fractures are the most common fractures of the upper extremity, with an incidence of 2 fractures per 1000 persons-years [1–3]. The past 25 years, an increase in open reduction and internal fixation (ORIF) for distal radius fractures has been observed [4, 5]. Especially, volar plating has increased in popularity [6, 7]. This approach provides better functional and radiological results compared to other surgical techniques [8–10]. Moreover, documented complications associated with dorsal plating and external fixation, and advancements in the locking plate technology have expanded the indications of volar plating [11]. However, to position the volar plate on the fracture site, the surgeon needs to detach the pronator quadratus muscle from its distal and radial side and lift it for optimal exposure to the fracture site [12]. Although the conventional approach involves repair of the pronator quadratus [12–14], controversy surrounds the merits of this repair. Some surgeons state the repair of the pronator quadratus will restore pronation strength and will protect the volar flexor tendons by covering the hardware [11, 15]. Conversely, other surgeons argue that the quality of the tissue often precludes a durable repair and a risk of ischaemic contracture of the pronator quadratus after tight closure exist, resulting in limited wrist pronation and supination [16, 17]. Despite these contradicting statements, 83% of the American hand surgeons repair the pronator quadratus following volar plate fixation [18].

Several studies evaluated the functional outcome and complications after volar locking plate fixation with and without pronator quadratus repair. A recent retrospective study of Hershman et al. found no differences between the two treatment groups, and they concluded that there was no advantage in repairing the pronator quadratus [19]. Another retrospective study of Ahsan et al. found no difference between complete and incomplete pronator quadratus repair [20]. However, regardless of their outcomes, they did recommend to make an effort to cover the volar plate with the pronator quadratus.

In conclusion, whether pronator quadratus repair after volar plate fixation is necessary remains a topic of debate. Therefore, the objective of this study was to systematically compile and evaluate the existing literature regarding pronator quadratus repair versus no pronator quadratus repair. To (1) compare the functional outcomes and (2) compare complication rates between pronator quadratus and no pronator quadratus repair after volar plate fixation.

## Methods

This systematic review was conducted according to the PRISMA checklist for reporting systematic reviews [21].

### Eligibility criteria

All studies comparing a group with pronator quadratus repair with a control group with no pronator quadratus repair in adult patients undergoing volar plate fixation for distal radius fractures, with a minimum follow-up of 12 months, were considered for inclusion. Studies comparing pronator quadratus repair with another repair or fixation technique were excluded. Moreover, case report and cadaveric studies were excluded. No restrictions regarding publication date or language were applied.

### Outcome measures

The primary outcome was patient-reported functional outcome, measured with the Disability of the Arm, Shoulder and Hand (DASH) score at 12 months follow-up. The DASH questionnaire is a validated self-report questionnaire, which measures patient’s physical function and symptoms [22].

Secondary outcomes included range of motion in terms of flexion, extension, pronation, supination, ulnar deviation and radial deviation, and grip strength measured with a dynamometer and as a percentage of the uninjured side. We also evaluated post-operative pain, indicated with the visual analogue scale (VAS) and the occurrence of complications. The documented complications of internal fixation of distal radius fractures include tendon injuries, neurovascular lesions, hardware failure, nonunion, malunion, carpal tunnel syndrome, compartment syndrome and deep and superficial infections [23].

### Literature search

To answer the research question, a comprehensive literature search was performed in Medline (Pubmed), EMBASE (Ovid) and Cochrane Central Register of Controlled Trials on 23 July 2015, with the assistance of a clinical librarian. The search strategy is depicted in Table 1. Additionally, a cross-reference check was performed.

**Table 1:** Search strategy

Pubmed: (“Radius Fractures”[Mesh] OR distal radius[tiab]) AND (pronator quadratus[tiab] OR pronat*[tiab] OR PQ[tiab] OR “Pronation”[Mesh]) AND (“Fracture Fixation, Internal”[Mesh] OR fixat*[tiab]) AND (“Volar Plate”[Mesh] OR volar[tiab] OR palmar[tiab]) EMBASE: (radius fracture/or distal radius.ti,ab,kw.) AND (body posture/or (pronator quadratus or pq).ti,ab,kw.) AND (osteosynthesis/or volar plate fixation/or (volar fixat* or palmar fixat*).ti,ab,kw.) Cochrane Central Register of Controlled Trials: #1 MeSH descriptor: [Radius Fractures] explode all trees #2 distal radius: ti,ab,kw (Word variations have been searched) #3 #1 or #2 #4 MeSH descriptor: [Pronation] explode all trees #5 pronator quadratus or pronat* or PQ:ti,ab,kw (Word variations have been searched) #6 #4 or #5 #7 MeSH descriptor: [Fracture Fixation, Internal] explode all trees #8 MeSH descriptor: [Volar Plate] explode all trees #9 volar or palmar or palmer:ti,ab,kw (Word variations have been searched) #6 #7 or #8 or #9 #11 #3 and #6 and #10 in Trials	

### Selection of eligible articles

Titles and abstract of all articles were screened independently by two reviewers, (MAMM and FJMEB), and either in- or excluded based on the in- and exclusion criteria. All duplicates were removed. Disagreement on relevance was addressed by discussion until a consensus was reached.

### Data extraction

Three reviewers extracted the data from the included studies independently. The extracted data included study type, number of included patients, patient characteristics, fracture characteristics according to the AO/OTA classification, details on the intervention and control group, length of follow-up and outcome measures.

The methodological quality of all included articles was assessed by two independent reviewers (MAMM and FJMEB) with the five-point Jadad score for randomised control trials [24] and the nine-point Newcastle–Ottawa Scale for non-randomised observational studies [25]. Level of evidence was provided in accordance with the Oxford Centre of Evidence-Based Medicine (http://www.cebm.net).

### Statistical analysis

Because of the paucity of the amount of studies regarding our research question and the difference in study designs, no pooling or meta-analyses was performed. Descriptive outcome analysis was used to compare the different results, data and outcomes between the studies.

## Results

### Study selection

**Fig. 1:**
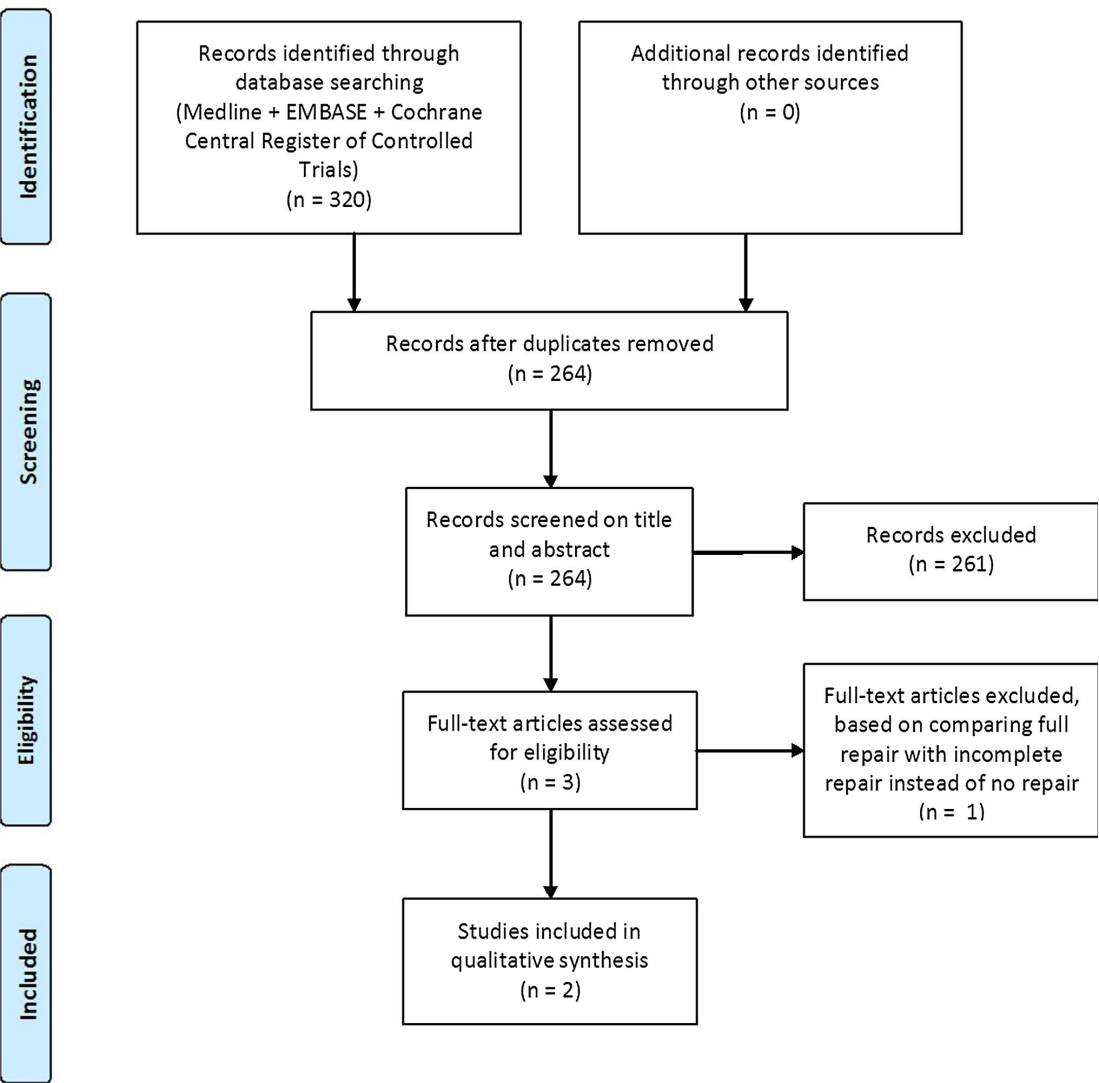
Flow diagram of study selection

A total of 320 articles were identified through our literature search. After removal of the duplicates 264 articles were selected. Two articles met our inclusion criteria and were included in this review. Only those patients who completed the 12 months follow-up were included. In total, 169 patients were included of which 95 underwent pronator quadratus repair and 74 patients had no pronator quadratus repair. The study selection process is summarised in Fig. 1.

### Included studies

The first study by Hershman et al. (2009) enrolled 175 patients undergoing open reduction and volar plate fixation of which 112 were included in this review because they completed the 12 months follow-up [19]. In this retrospective study, patients were included from a prospective database in which they were enrolled over a 5-year period (2004–2009). In 62 patients (mean age 53.8 years), the pronator quadratus was repaired during volar plating fixation (intervention group) and 50 patients (mean age 51.6 years) had no pronator quadratus repair (control group). The choice whether or not to repair the pronator quadratus was surgeon dependent. All surgeries in the intervention group were performed by a fellowship trained hand surgeon with volar plates from Depuy Orthopedics (Warsaw, IN, USA). The surgeries in the control group were performed by a fellowship trained orthopaedic trauma surgeon using volar plates from Stryker (Mahwah, NJ, USA). Evaluators were not blinded to the treatment but unaware of surgical details. Measurements were taken by one of several independent, trained research assistants. Range of motion and grip strength was documented as a percentage of the uninjured arm and measured, respectively, with a goniometer and handgrip dynamometer. Only the outcomes at final follow-up, at 12 months, were presented.

Tosti et al. (2013) conducted a double blind, prospective, randomised clinical trial from January 2011 to December 2011 [16]. Sixty patients with distal radius fractures were assigned to the intervention group or the control group based on their year of birth. Patients born in an odd birth year were assigned to the intervention group, patients born in an even birth year were assigned to the control group. All patients were blinded to their respective study group. An orthopaedic nurse, blinded to the study protocol, obtained all of the outcome measurements. Range of motion was measured in degrees with a goniometer, and grip strength was measured with a dynamometer. Both range of motion and grip strength were presented as a percentage of the uninjured side. All patients were assessed at 2 and 6 weeks and at 3 and 12 months. Full follow-up data was available for 57 patients. Three patients were excluded from the study.

Patients of both studies were comparable regarding age. The study of Hershman et al. included a higher percentage of man compared to Tosti et al. Also, the study of Tosti et al. included a higher percentage of AO/OTA type C fractures compared to Hershman et al. The study characteristics of both studies are summarised in Table 2.

**Table 2:** Study characteristics

	Study design	Total number of patients	Total number of included patients with complete follow-up		Mean age (years)	Male patients (%)	Injury to dominant hand (%)	AO/OTA classification (%)		
			PQ repair	No PQ repair				*A*	*B*	*C*
Hershman et al. (2009)	Prospective database, retrospectively reviewed	175	62	50	52.8	45	41	35.7	19.6	44.6
Tosti et al. (2013)	Randomised controlled trial	60	33	24	57.9	26	58	17.5	3.5	79

*PQ* pronator quadratus

### Quality assessment of included studies

The methodological quality of the only randomised trial (Tosti et al. 2013) scored 3 points out of the maximum of 5 points on the Jadad Scale. The study of Hershman et al. (2009) was assessed using the Newcastle–Ottawa Scale; scoring 6 points out of the maximum of 8 points.

### Functional outcome

At 12 months follow-up no statistically significant differences in DASH scores were observed between the repair and the no repair group (Hershman et al. 16.2 (95% CI 11.8–20.6), respectively, 11.2 (95% CI 7.2–15.2) and Tosti et al. 8, respectively, 5). Moreover, no significant differences in pronation or supination were found.

Hershman et al. found a trend towards improved DASH scores (*p* = 0.10), pronation (*p* = 0.08), grip strength (*p* = 0.12) and pain (*p* = 0.13) in patients undergoing no pronator quadratus repair; however, this was not statistically significant. However, a statistically significant difference in radial deviation in favour of the no repair group was found (Table 3).

**Table 3:** Functional outcomes at 12 months follow-up

	Hershman et al. (2009)			Tosti et al. (2013)		
	Repair group	No repair group	*p* value	Repair group	No repair group	*p* value
Mean DASH-score	16.2	11.2	n.s.	8	5	n.s.
Mean VAS score	1.83	1.20	n.s.	<0.5	<0.5	n.s.
Grip strength (% contralateral)	78.6%	104.5%	n.s.	95%	95%	n.s.
*Range of motion:*						
Extension (% contralateral or degrees)	85.0%	87.1%	n.s.	83°	80°	n.s.
Flexion (% contralateral or degrees)	85.1%	91.1%	n.s.	84°	81°	n.s.
Pronation (% contralateral of degrees)	97.1%	101%	n.s.	84°	84°	n.s.
Supination (% contralateral or degrees)	93.2%	98.7%	n.s.	88°	86°	n.s.
Ulnar deviation (% contralateral or degrees)	89.4%	85.5%	n.s.	36°	35°	n.s.
Radial deviation (% contralateral or degrees)	76.7%	100.5%	0.03	19°	20°	n.s.

*n.s.* not significant

Tosti et al. also determined the assessed outcomes at 2 and 6 weeks, and 3 months. Outcomes assessed at 2 weeks and 3 months demonstrated no significant differences in mean DASH score, VAS, grip strength and range of motion. However, at 6 weeks, grip strength and flexion in the repair group were significantly better compared to the no repair group, but all other variables were not significantly different.

Overall, the mean values of all variables demonstrated a stepwise improvement over the year as range of motion, grip strength consistently increased, and DASH and VAS scores consistently decreased.

### Complications

In total seven patients underwent plate removal, five in the repair group and two in the no repair group. Plate removal was performed for several reasons including flexor or extensor tendon irritation due to a prominent hardware, intra-articular penetration of screws or complaints in the context of carpal tunnel syndrome. Only Hershman et al. reported two cases of extensor pollicis longus tendon rupture, one in each group. Both were detected at 6 weeks follow-up. No deep or superficial wound infections or nonunions were observed (Table 4).

**Table 4:** Complications at 12 months follow-up

	Repair group	No repair group
Wound infections (deep and superficial)	0	0
Nonunion/malunion	0	0
Tendon irritation/tenosynovitis	2	1
EPL tendon rupture	1	1
Hardware removal	5	2
Carpal tunnel syndrome	3	0

*EPL* extensor pollicis longus

## Discussion

With the results of this systematic review of existing literature we found no evidence that pronator quadratus repair after volar plate fixation in distal radius fractures provides better functional outcomes. DASH scores, range of motion, post-operative pain and minor complications are similar in patients who undergo pronator quadratus repair to patients who do not undergo pronator quadratus repair.

Repairing the pronator quadratus theoretically protects the flexor tendons against the volar plate and sharp edges of the screw heads and serves as a dynamic stabilizer of the distal radioulnar joint (DRUJ) [17, 26, 27]. Although Swigart et al. stated that pronator quadratus repair after volar plate fracture fixation is often durable [18], repair could also be suboptimal due to the very short tendon or a damaged and friable muscle, that renders it unsuitable for suturing [28]. Moreover, the integrity of the muscle is not checked after the repair to assess if the repaired pronator quadratus is still in place or has not detached again. Additionally, repair of the pronator quadratus muscle does not seem to adequately protect the tendons, since cases of late tendon irritation after pronator quadratus repair are still identified [29], and flexor pollicis longus rupture in particular is caused by a too distal positioning of the plate, prominent at the watershed line, which may increase the risk of tendon injury [27, 30, 31].

From previous studies we know that the pronator quadratus can be divided into a superficial head, which is the primary contributor to forearm pronation, and a deep head, which is the dynamic stabilizer of the distal radioulnar joint [32, 33]. Although, the superficial head is the one that is repaired or not, pronation strength from the deep head should be maintained. Studies performed to assess the contribution of the pronator quadratus to pronation torque of the forearm state that the pronator quadratus is the primary pronating muscle in the forearm [26, 34, 35] and that significant decrease in pronation torque occurs with controlled elimination of the pronator quadratus function [34, 36]. However, those studies were either performed in small populations or complete paralysation of the pronator quadratus was reached by injecting lidocaine into the muscle.

In contrast, two other larger studies found no difference in pronation strength between the operated and the healthy wrist and isokinetic forearm rotation strength and the length of the healed pronator quadratus [37, 38]. Additionally, Häberle et al. found no differences in isometric pronation strength between pronator quadratus repair and no pronator quadratus repair after volar plate fixation [39]. This is consistent with our findings which show no significant differences in pronation or supination of the forearm. Moreover, a retrospective study performed by Ahsan et al. that compared full pronator quadratus repair with incomplete pronator quadratus repair [20] did not find a significant difference in pronation and supination, as well as grip strength and post-operative complications either.

The only significant difference found by Hershman et al. was a 23.8% difference, compared to the contralateral uninjured wrist, in radial deviation in favour of no pronator quadratus repair at 12 months follow-up. Tosti et al. found a significant difference at 6 weeks follow-up in grip strength and flexion in favour of pronator quadratus repair. However, we cannot ascribe these differences to the pathomechanics of the incised pronator quadratus.

Recently, a new technique was introduced; minimally invasive plate osteosynthesis. With this new technique the pronator quadratus muscle will only undergo a minimal elevation to create a pocket between the surface of the distal radius and the pronator quadratus muscle where the volar plate can be inserted. The aim of this new technique is to reach a better fracture healing process by maintaining the blood supply in the pronator quadratus muscle, preserving pronator quadratus function and preventing tendon injury [40–43]. A retrospective study in 66 patients done by Zenke et al. compared conventional volar plate fixation with minimally invasive plate osteosynthesis [42]. However, they found no significant differences between both groups in DASH scores, range of motion, grip strength and VAS-scores. In addition, there were no significant differences in fracture healing between both groups and therefore the fracture repair process was not obviously augmented by keeping the pronator quadratus muscle and the blood supply intact.

This systematic review has some limitations. Only two studies met our inclusion criteria, of which only one randomised controlled trial, and therefore our results incorporate a limited number of patients (235 of whom 169 (72%) completed a 12 months follow-up). Moreover, due to the two different study designs and the different units of the outcome variables, we were not able to pool our data. To give a unambigious conclusion on the fact if repair of the pronator quadratus after volar plate fixation is necessary, more prospective randomized trials with the same units of the outcome variables are needed.

## Conclusion

In this systematic review we found no statistically significant differences regarding functional outcomes, range of motion, grip strength, post-operative pain and complications between repair of the pronator quadratus muscle after volar plate fixation and no repair. Moreover, the quality and durability of the repair are questionable. Also no statistically significant differences in functional outcome are seen between minimally invasive plate osteosynthesis, where the pronator quadratus is preserved, and conventional volar plate fixation. Based on these results, we do not see any advantages of pronator quadratus repair after volar plate fixation in the distal radius. However, a definitive conclusion on the fact whether repair of the pronator quadratus after volar plate fixation is necessary cannot be drawn from this systematic review. Therefore, more prospective randomized trials with the same units of the outcome variables would be necessary. Additionally, these studies should take into account the quality and durability of the pronator quadratus repair.
